# The relationship between personality traits and job satisfaction among physicians at designated infectious disease medical institutions in Gansu province: the mediating role of negative coping strategies

**DOI:** 10.1186/s12913-026-14204-y

**Published:** 2026-03-25

**Authors:** Sheng Li, Zhiguo Li, Yujie Wu, Yuting Li, Jinyu Wang, Liping Liang

**Affiliations:** 1 The No. 2 People’s Hospital of Lanzhou, Lanzhou City, 730046 China; 2 School of Public Health, Gansu University of Chinese Medicine, Lanzhou City, 730101 China; 3 First Clinical Medical College, Gansu University of Chinese Medicine, Lanzhou City, 730101 China; 4 School of Public Health, Lanzhou University, Lanzhou City, 730000 China; 5 Wuwei Center for Disease Control and Prevention, Wuwei City, Gansu Province 733000 China

**Keywords:** Personality traits, Negative coping, Job satisfaction, Physicians, Structural equation modeling

## Abstract

**Objective:**

To examine the relationship between personality traits and job satisfaction among physicians working in designated infectious disease medical institutions in Gansu Province, and to clarify the mediating role of negative coping strategies in this association. The study aims to deepen the understanding of psychological mechanisms underlying physicians’ job satisfaction in high-risk medical settings and to provide evidence-based guidance for targeted management and intervention strategies.

**Method:**

A cross-sectional questionnaire survey was conducted among physicians employed at designated infectious disease medical institutions in Gansu Province. Data on personality traits, negative coping strategies, and job satisfaction were collected, and structural equation modeling was used to analyze the direct and indirect relationships among these variables.

**Results:**

Neuroticism exhibits a negative influence on job satisfaction, whereas conscientiousness, agreeableness, openness, and extraversion demonstrate a positive impact on job satisfaction.Negative coping styles exert a detrimental influence on physicians’ job satisfaction. Neuroticism among physicians positively correlates with negative coping styles, whilst conscientiousness, agreeableness, openness and extraversion exhibit negative correlations with such coping styles.Passive coping mediates the relationship between doctors’ personality traits and job satisfaction.

**Conclusion:**

Personality traits influence physicians’ job satisfaction both directly and indirectly through coping strategies, with negative coping playing a key mediating role. These findings suggest that interventions targeting coping styles, together with organizational support tailored to individual personality characteristics, may be effective approaches to improving physicians’ job satisfaction and professional well-being in designated infectious disease medical institutions.

## Introduction

In recent years, as occupational risks and work intensity within the healthcare system have continued to rise, the mental health and job satisfaction of medical personnel have become critical factors influencing staff turnover and healthcare quality.Extensive research worldwide and in China consistently reports that both physician burnout levels and job satisfaction exhibited significant fluctuations before and after the COVID-19 pandemic: During the peak of the outbreak, heightened emotional exhaustion, heavy workloads, and uncertainty markedly reduced healthcare workers’ job satisfaction. In the later stages of the pandemic, a complex situation emerged across regions characterized by “partial recovery coexisting with persistently high stress.” These phenomena collectively indicate that external environmental stressors exert a direct and significant impact on the psychological state of healthcare workers [1, 2].

In previous research, job satisfaction has typically been regarded as jointly determined by external factors such as organisational support, workload, remuneration systems, and career development [3]. However, explaining variations in healthcare professionals’ job satisfaction solely through environmental factors fails to adequately account for individual differences. Personality traits, as relatively stable psychological and behavioural tendencies, play a significant role in stress perception, emotional regulation, and occupational adaptation. Based on the Big Five personality theory, neuroticism, extraversion, agreeableness, conscientiousness, and openness constitute the core dimensions describing individual personality differences. Existing research indicates that individuals with higher neuroticism levels are more prone to emotional instability and negative evaluations, thereby showing a significant correlation with lower job satisfaction [4]. In contrast, high agreeableness and conscientiousness are often associated with better work adaptation and higher occupational satisfaction. Physicians with high openness are better equipped to proactively adapt to change and embrace novel experiences (such as new technologies or concepts), thereby reconstructing meaning in stressful situations and enhancing job satisfaction [5]. Extraversion is closely linked to positive affective experiences. Extroverted individuals exhibit stronger positive emotional responses to rewards and social events or activities [6]. However, existing research predominantly focuses on general healthcare settings or broad occupational groups, with insufficient attention given to physicians in designated infectious disease facilities operating within high-risk, high-exposure environments. Based on the aforementioned theoretical framework and literature, the following hypothesis is proposed:

H1: Neuroticism exhibits a negative influence on job satisfaction, whereas conscientiousness, agreeableness, openness, and extraversion demonstrate a positive impact on job satisfaction.

Psychological stress theory posits that when individuals encounter external stressors, their psychological and behavioural responses are not solely determined by the stressful event itself, but are modulated by the individual’s cognitive appraisal and coping strategies. Coping mechanisms, serving as crucial psychological links between personality traits and stress responses, are typically categorised into two types: positive coping and negative coping. Positive coping emphasises problem-solving and resource integration, aiding stress alleviation and maintaining psychological well-being. Conversely, negative coping (such as avoidance, denial, or emotional reactivity) may lead to accumulated stress and impaired psychological functioning. Substantial evidence indicates [7, 8] that individuals employing negative coping strategies develop pessimistic thoughts and evasive behaviours, doubt their professional capabilities, and experience diminished occupational quality of life, thereby increasing the risk of reduced job satisfaction. Furthermore, coping style selection is significantly influenced by personality traits [9]. Research indicates [10] that nurses with high neuroticism are more prone to negative emotions and exhibit poor emotional regulation in high-pressure work environments. Conversely, individuals scoring higher on conscientiousness, agreeableness, extraversion, and openness tend to adopt proactive coping strategies. Based on the aforementioned theories and literature, the following hypothesis is proposed:

H2: Negative coping styles exert a detrimental influence on physicians’ job satisfaction.

H3: Neuroticism among physicians positively correlates with negative coping styles, whilst conscientiousness, agreeableness, openness and extraversion exhibit negative correlations with such coping styles.

The integrated theory of personality traits and the stress-coping model suggests that personality characteristics not only directly influence job satisfaction but may also exert indirect effects through psychological mechanisms such as coping styles. Research indicates that the pathway linking personality to job fit involves both direct effects and mediation through variables such as coping styles, psychological capital, and social support [11]. However, within the high-stress, high-risk occupational context of infectious disease designated medical institutions, the mediating role of negative coping between physicians’ personality traits and job satisfaction—specifically its direction and effect strength—remains under-examined. Based on the aforementioned theories and literature, the following hypothesis is proposed:

H4: Passive coping mediates the relationship between doctors’ personality traits and job satisfaction.

Given this context, the present study systematically examines the direct influence of personality traits on job satisfaction among physicians at designated infectious disease treatment institutions in Gansu Province, alongside the mediating role of negative coping strategies. Employing a cross-sectional survey methodology grounded in the Big Five personality theory and psychological stress-coping model, structural equation modelling was utilised for analysis. This study aims to deepen understanding of the pathways influencing healthcare professionals’ occupational psychology. It seeks to provide data support for enhancing physicians’ professional experiences, developing targeted psychological support strategies, and promoting the scientific allocation of healthcare human resources. Furthermore, it offers theoretical foundations and practical guidance for advancing the stability and sustainable development of public health systems.

## Methodology

### Research subjects and data collection

This study was conducted between 27 March and 20 April 2024. Through coordination by the Gansu Provincial Health Commission, the research team obtained a list of all designated infectious disease treatment institutions across the province. Sample selection followed this procedure: first, research invitation letters and protocol descriptions were sent to the administrative departments of all target institutions; upon obtaining institutional consent, designated liaisons from each institution distributed electronic questionnaire links to all eligible physician work groups. A total of 8,129 questionnaires were distributed. After excluding invalid responses, 8,071 valid data points were retained, yielding a net response rate of approximately 99.28%. Inclusion criteria specified practising physicians with ≥ 1 year of clinical experience in infectious diseases. Exclusion criteria comprised: ① medical personnel undergoing advanced training; ② registered staff on continuous leave exceeding three months; ③ Interns and medical personnel undertaking specialised external training. This research protocol has been approved by the Medical Ethics Review Committee of Lanzhou Pulmonary Hospital (No: 2024111901) and informed consent has been obtained from all participants. The questionnaire cover page detailed the research objectives, confidentiality principles, voluntary participation, and the right to withdraw at any time. An electronic informed consent option was included, requiring participants to tick ‘Agree’ before accessing the main questionnaire. Encrypted questionnaire links were distributed via designated institutional liaisons. The questionnaire was configured to permit only one submission per device or IP address, ensuring independent responses. Participants completed the anonymous survey during their personal time via mobile phone or computer, with an average completion time of 10–18 min.

### Measurement

#### Demographic characteristics questionnaire

Content includes: gender, age, title, monthly income, highest Education Level, years of experience, hobbies and Interests.The aforementioned variables are closely associated with physicians’ job satisfaction; consequently, they were incorporated as control variables in the subsequent structural equation modelling to mitigate potential confounding effects.

#### Chinese big five personality inventory brief version (CBF–PI–B)

The scale [12] comprises 40 items assessing five dimensions: Neuroticism, Conscientiousness, Agreeableness, Extraversion, and Openness. Each dimension contains 8 items rated on a 6-point Likert scale (1 = Strongly disagree, 2 = Mostly disagree, 3 = Somewhat disagree, 4 = Somewhat agree, 5 = Mostly agree, 6 = Strongly agree). This scale includes seven reverse-scored items (Items 5, 8, 13, 15, 18, 32, and 36), requiring score conversion according to reverse-scoring rules. In this study, the overall Cronbach’s α for the scale was 0.915.

#### Simplified coping style questionnaire (SCSQ)

The questionnaire [13] comprises 20 items, with items 13–20 assigned to the “negative coping” dimension. It employs a 4-point Likert scale (0 = never use, 1 = rarely use, 2 = sometimes use, 3 = often use). Higher scores on the negative coping dimension indicate a greater tendency toward negative coping strategies. In this study, the positive coping dimension achieved a Cronbach’s α of 0.835.

#### Minnesota satisfaction questionnaire (MSQ) short form

The short-form MSQ [14] comprises 20 items reflecting intrinsic satisfaction, extrinsic satisfaction, and overall job satisfaction (items 17 and 18 assess general job satisfaction). Extrinsic satisfaction comprises items 5, 6, 12, 13, 14, and 19, while intrinsic satisfaction includes items 1–4, 7–11, 15, 16, and 20. A 5-point Likert scale is used (1 = Very dissatisfied, 2 = Dissatisfied, 3 = Undecided, 4 = Satisfied, 5 = Very satisfied), with higher scores indicating greater job satisfaction. Cronbach’s α for this scale in the present study was 0.965.

### Quality control

To ensure data quality, this study strictly adhered to standardized operating procedures: all investigators completed pre-service systematic training and passed qualification assessments; encrypted electronic platforms were used for anonymous questionnaire distribution and data collection to ensure independent respondent responses and protect privacy. Data cleaning employed a three-tier screening mechanism: ① Exclusion of duplicate responses; ② Elimination of questionnaires with ≥ 3 missing items or obvious logical inconsistencies; ③ Removal of questionnaires exhibiting identical response patterns across all dimensions, suspected to be invalid.

### Common method bias

Given that this study employed self-administered questionnaires for data collection, common method bias may be present. To address this, Harman’s single-factor test was applied for diagnostic purposes. Results indicate that the variance explained by the first common factor extracted prior to rotation was 37.06%, falling below the critical threshold of 40.00%. This suggests that common method bias does not pose a significant threat to data quality, permitting subsequent analyses to proceed.

### Statistical methods

All statistical analyses were performed using SPSS 26.0 and AMOS 24.0. First, the K-S test confirmed the data distribution. Results indicated non-normal distribution, thus quantitative data are presented as median (interquartile range) [M(P25, P75)]. Pairwise comparisons employed the Wilcoxon signed-rank test, while multiple comparisons used the Kruskal–Wallis H test; Spearman’s correlation coefficient assessed relationships between variables. In model analysis, structural equation modeling (SEM) was employed to construct theoretical pathways, with parameter estimation conducted via maximum likelihood estimation. Bootstrap resampling (5000 iterations) was used to estimate direct effects, mediating effects, and total effects, calculating 95% bias-corrected confidence intervals (95% *CI*). An effect was deemed statistically significant when its confidence interval excluded zero and *P* < 0.05.

## Results

### Distribution of job satisfaction across demographic characteristics

Statistically significant differences in job satisfaction were observed across gender, age, professional title, monthly income, highest educational attainment, years of service, and hobbies (*P* < 0.05). See Table 1.

**Table 1:** Work satisfaction scores of physicians at designated infectious disease medical institutions by demographic Characteristics(*n* = 8071)

Variable	Frequency	Job Satisfaction Score	Z/H	*P*
Gender				
Man	3616	70(60,80)	Z=-6.062	< 0.01
Woman	4455	73(61,80)		
Age				
≤ 30	2608	72(60,80)^a^	H = 20.973	< 0.01
31∼40^c^	2885	70(60,79)		
41∼50	1685	72(60,80)^a^		
>50	893	73(62,80)^a^		
Title				
Junior and below	4442	72(60,80)^ab^	H = 28.686	< 0.01
middle level^c^	2125	70(60,79)		
deputy high level	1206	71(60,80)^b^		
high level^d^	298	75(64,80)^a^		
Monthly Income(¥)				
<3,000	1575	69(60,80)^a^	H = 41.966	< 0.01
3,000–6,000^c^	5299	71(60,80)^b^		
>6,000^d^	1197	74(63,80)^a^		
Highest Education Level				
College diploma or below	1393	74(61,80)^a^	H = 28.818	< 0.01
Bachelor’s degree^c^	6441	71(60,80)		
Master’s degree	232	73(60,80)		
Doctoral degree	5	78(52.5,80)		
Years of Experience				
≤ 3	1286	75(60,80)^ab^	H = 46.899	< 0.01
3.1∼10^c^	2882	70(60,80)		
10.1∼25^d^	2670	71(60,80)		
>25	1233	73(62,80)^ab^		
Hobbies and Interests				
None^c^	1825	68(60,79)	H = 76.139	< 0.01
1–2	5161	72(61,80)^a^		
> 2	1085	73(60,80)^a^		

Note: Compared with c, a *P* < 0.05; compared with d, b *P* < 0.05

### Correlation between personality traits, negative coping strategies, and job satisfaction

Spearman correlation analysis was conducted on the personality traits, negative coping strategies, and job satisfaction of physicians at designated infectious disease hospitals. The scores for personality traits, negative coping strategies, and job satisfaction among survey participants were correlated. Results indicate statistically significant associations among personality traits, negative coping strategies, and job satisfaction (*P* < 0.05), as shown in Table 2.

**Table 2:** Correlation analysis of personality traits, negative coping and job satisfaction scores of medical staff in designated hospitals in Gansu Province

Variable	Neuroticism	Conscientiousness	Agreeableness	Openness	Extraversion	Negative Coping	Job Satisfaction
Neuroticism	1						
Conscientiousness	−0.127^**^	1					
Agreeableness	−0.199^**^	0.708^**^	1				
Openness	−0.01	0.498^**^	0.373^**^	1			
Extraversion	−0.332^**^	−0.250^**^	−0.189^**^	−0.055^**^	1		
Negative Coping	0.172^**^	−0.132^**^	−0.134^**^	−0.140^**^	−0.067^**^	1	
Job Satisfaction	−0.264^**^	0.398^**^	0.382^**^	0.308^**^	0.042^**^	0.430^**^	1

Note: ** *p* < 0.01

### Structural equation model of personality traits, negative coping strategies, and job satisfaction

**Fig. 1:**
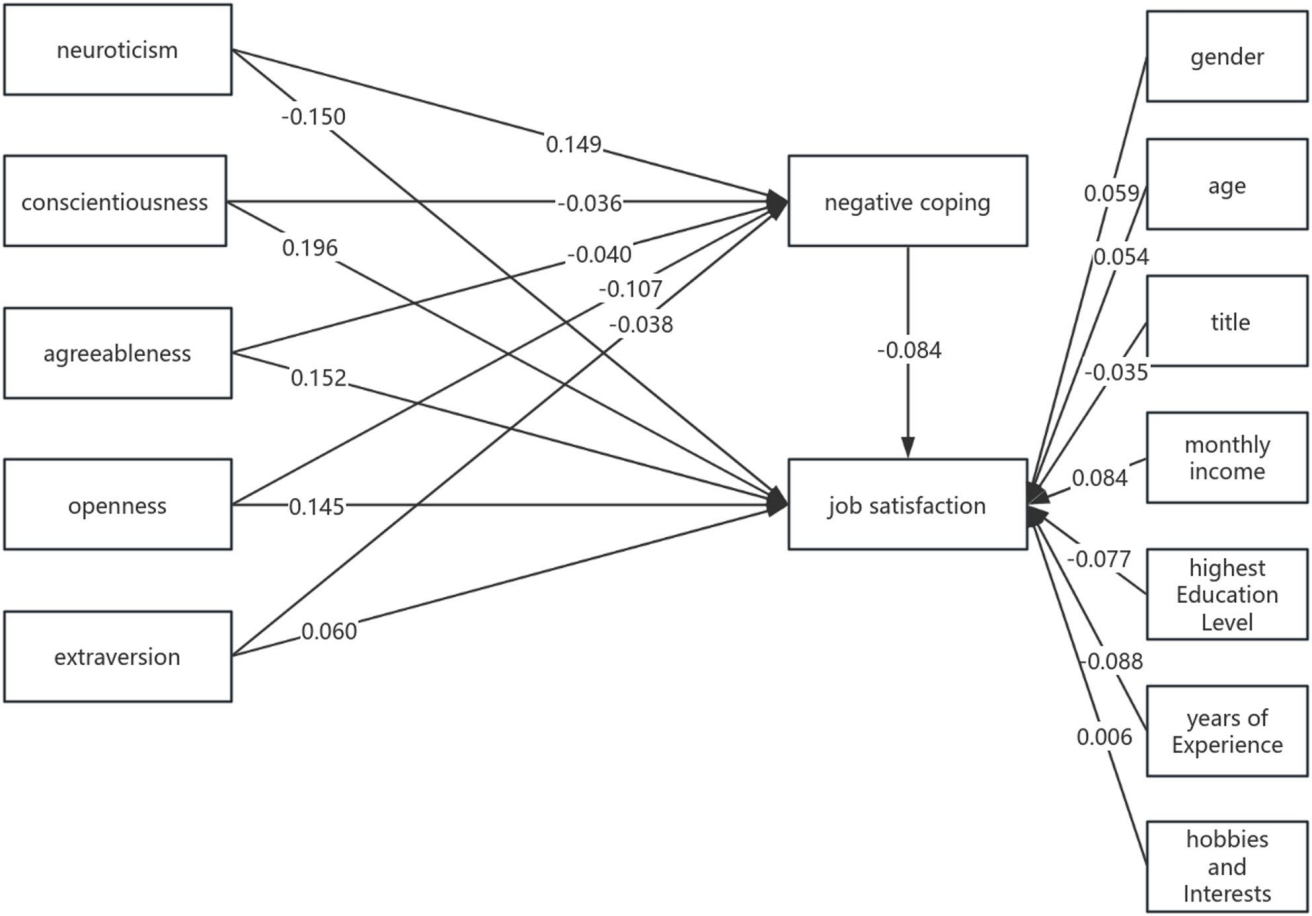
Structural equation model of personality traits, negative coping, and job satisfaction

Based on psychological stress theory, a path model was constructed to illustrate how physicians at designated infectious disease medical institutions experience work satisfaction under various stressors (influencing factors), mediated by the amplifying or attenuating effects of positive coping strategies. Concurrently, variables yielding significant results in single-factor analyses were incorporated as control variables within the structural equation model to mitigate potential confounding effects.Model fit was assessed using maximum likelihood estimation. After iterative model refinement, path coefficients were estimated, with results presented in Fig. 1. Overall model fit was evaluated primarily using fit indices: χ^2^/DF = 3.309, CFI = 0.999, TLI = 0.994, IFI = 0.999, GFI = 0.999, AGFI = 0.994 (all > 0.9), and RMSEA = 0.017 (< 0.05). All indicators fell within acceptable ranges.

### Structural path relationships among personality traits, negative coping, and job satisfaction

As shown in Path Results Fig. 1, various personality traits (dependent variables) primarily influence job satisfaction among physicians at designated infectious disease medical institutions through the following mediating variable, “negative coping,” either directly or indirectly. The path results indicate: Negative coping has a negative impact on doctors’ job satisfaction, thus supporting Hypothesis H2; Neuroticism has a positive impact on negative coping, while Conscientiousness, Agreeableness, Openness, and Extraversion have a negative impact on negative coping, thus supporting Hypothesis H3. To further illustrate the effect sizes among variables, the bias-corrected Bootstrap method was employed with 5,000 samples, calculating the average coefficient from 5,000 runs. When negative coping serves as a mediating variable, neuroticism, conscientiousness, agreeableness, openness, and extraversion may exert indirect effects on job satisfaction by influencing negative coping strategies, thus supporting Hypothesis H4. See Tables 3 and 4.

**Table 3:** Path relationships in the structural equation model

Path	SE	Estimate	S.E.	C.R.	*P*
gender→Job Satisfaction	0.059	1.723	0.293	5.886	***
age→Job Satisfaction	0.054	0.802	0.286	2.8	0.005
title→Job Satisfaction	-0.035	-0.599	0.261	-2.298	0.022
monthly income→Job Satisfaction	0.084	2.105	0.293	7.187	***
highest Education Level→Job Satisfaction	-0.077	-2.619	0.359	-7.301	***
years of Experience→Job Satisfaction	-0.088	-1.372	0.285	-4.818	***
hobbies and Interests→Job Satisfaction	0.006	0.138	0.242	0.571	0.568
Neuroticism→Negative Coping	0.149	0.080	0.006	12.317	***
Conscientiousness→Negative Coping	-0.036	-0.020	0.010	-2.006	0.045
Agreeableness→Negative Coping	-0.040	-0.024	0.010	-2.468	0.014
Openness→Negative Coping	-0.107	-0.074	0.009	-7.992	***
Extraversion→Negative Coping	-0.038	-0.039	0.013	-3.082	0.002
Negative Coping→Job Satisfaction	-0.084	-0.311	0.037	-8.501	***
Neuroticism→Job Satisfaction	-0.150	-0.296	0.021	-13.802	***
Conscientiousness→Job Satisfaction	0.196	0.393	0.032	12.347	***
Agreeableness→Job Satisfaction	0.152	0.349	0.033	10.652	***
Openness→Job Satisfaction	0.145	0.368	0.031	12.004	***
Extraversion→Job Satisfaction	0.060	0.223	0.041	5.429	***

Note: *** *p* < 0.001

**Table 4:** Mediation effect analysis of paths in the structural equation model

Path	Effects	SE	S.E.	P值	95%CI	
					LB	UB
Neuroticism→Negative Coping→Job Satisfaction	Overall Effects	-0.162	0.013	< 0.001	-0.188	-0.137
	Direct Effects	-0.149	0.013	< 0.001	-0.175	-0.124
	Indirect Effects	-0.013	0.002	< 0.001	-0.017	-0.09
Conscientiousness→Negative Coping→Job Satisfaction	Overall Effects	0.199	0.018	< 0.001	0.164	0.234
	Direct Effects	0.196	0.018	< 0.001	0.162	0.231
	Indirect Effects	0.003	0.002	0.049	0.001	0.006
Agreeableness→Negative Coping→Job Satisfaction	Overall Effects	0.156	0.016	< 0.001	0.124	0.185
	Direct Effects	0.152	0.016	< 0.001	0.121	0.182
	Indirect Effects	0.004	0.001	0.014	0.001	0.006
Openness→Negative Coping→Job Satisfaction	Overall Effects	0.154	0.013	< 0.001	0.129	0.179
	Direct Effects	0.145	0.013	< 0.001	0.120	0.170
	Indirect Effects	0.009	0.002	< 0.001	0.006	0.013
Extraversion→Negative Coping→Job Satisfaction	Overall Effects	0.063	0.011	< 0.001	0.040	0.084
	Direct Effects	0.060	0.011	< 0.001	0.037	0.081
	Indirect Effects	0.003	0.001	0.001	0.001	0.006

## Discussion

This study, grounded in psychological stress theory and structural equation modeling, examines the influence mechanism of personality traits on job satisfaction among physicians at designated infectious disease treatment facilities, mediated by negative coping styles. Through questionnaire surveys and data analysis of physicians from multiple designated hospitals in Gansu Province, we found that personality traits not only directly impact job satisfaction but also exert indirect effects via negative coping styles. This further illuminates the complex relationship between healthcare workers’ mental health and professional behavior.

### Demographic variations in job satisfaction: combined effects of career stage and organizational factors

The findings of this study indicate that demographic characteristics such as gender, age, professional title, monthly income, and educational attainment among physicians at designated infectious disease treatment centres are significantly correlated with their job satisfaction. These disparities do not exist in isolation but are closely linked to the stage of career development in which individuals find themselves, reflecting the dynamic shifts in core needs, professional challenges, and resource acquisition capabilities faced by physicians at different career junctures. From a career development theory perspective, job satisfaction functions as a measure of the degree to which personal needs align with workplace provision, a match that undergoes systematic evolution throughout one’s professional journey [15]. For instance, junior doctors may prioritise skill acquisition and career advancement opportunities, whereas senior practitioners may place greater emphasis on professional autonomy and occupational identity. This shift in demand focus directly influences their satisfaction ratings across different aspects of their work [16].

Specifically, female physicians exhibit higher job satisfaction than their male counterparts, a trend consistent with previous literature [17]. This may stem from women demonstrating greater proficiency in patient communication, emotional attentiveness, and time investment [18], thereby gaining more positive experiences in organizational support and emotional rewards. Regarding age and tenure, physician satisfaction exhibits a typical “mid-career dip” pattern: satisfaction is lowest among those aged 31–40 and with 3.1–10 years of experience, while satisfaction is relatively higher among those aged ≤ 30 and ≥ 50, as well as those with ≤ 3 years and ≥ 25 years of experience. This finding aligns with international research on healthcare professionals’ career lifecycle patterns, where the middle-age stage often involves multiple pressures such as work stress and increased family responsibilities, making individuals prone to occupational burnout and decreased satisfaction [19]. Regarding professional title and monthly income, satisfaction levels were significantly higher in the high-title and high-income groups compared to other groups. This indicates that career development opportunities and economic rewards remain core factors influencing physicians’ professional well-being. Higher professional titles correlate with increased income and benefits, reducing work and promotion pressures compared to lower-title physicians, thereby enhancing job satisfaction [20]. Particularly in infectious disease settings, where high occupational risks and frequent exposure are prevalent, appropriate compensation incentives and clear promotion pathways significantly boost physicians’ organizational commitment. Regarding highest educational attainment, the group with the highest level of education demonstrated significantly higher job satisfaction than other groups. Physicians with advanced degrees typically possess stronger professional and research capabilities. Their job responsibilities and professional roles align closely with their educational background, offering more promotion opportunities, greater professional autonomy, and more substantial compensation incentives. This leads to higher job-person fit and greater professional fulfillment. Notably, differences in hobbies and interests also showed a significant correlation with satisfaction. Physicians lacking recreational activities reported the lowest satisfaction, while those with one to two or multiple hobbies demonstrated higher satisfaction. This primarily stems from leisure activities promoting physical and mental recovery, alleviating burnout from prolonged high-pressure environments. These findings further underscore the importance of fostering work-life balance within high-risk departments.

### Direct effects of personality traits on job satisfaction

The path analysis results of this study clearly indicate that each dimension of the Big Five personality has a significant direct effect on job satisfaction. Neuroticism negatively predicts job satisfaction, while Conscientiousness, Agreeableness, Openness, and Extraversion positively predict job satisfaction. Among Iranian nurses during the COVID-19 pandemic, neuroticism and conscientiousness were also found to positively influence job satisfaction [21]. Results indicate that conscientiousness exhibits the strongest direct positive effect (effect size = 0.194), aligning closely with the core tenets of this trait. Highly conscientious physicians typically demonstrate strong organizational skills, a sense of responsibility, and achievement orientation. Little is known about how conscientiousness leads to negative outcomes [22]. In highly rigorous and standardized work environments like infectious disease control, these traits effectively translate into exceptional performance and lower error rates, fostering strong professional accomplishment and satisfaction. Additionally, agreeableness and openness also demonstrated strong positive predictive power. Physicians with high agreeableness are more inclined to foster harmonious doctor-patient relationships and team collaboration. Existing research indicates that agreeableness is significantly correlated with empathy, communication quality, prosocial behavior, and higher patient satisfaction, while also playing a positive role in healthcare team cooperation and job satisfaction [23–25]. Physicians with high openness demonstrate greater adaptability and learning capacity toward new treatment protocols, technical procedures, and unexpected situations. Literature indicates openness is closely linked to receptivity to new knowledge, flexibility in clinical decision-making, and stronger coping and learning abilities in complex scenarios [26, 27], enabling them to more proactively address professional challenges.

In contrast, neuroticism exhibits a significant negative direct effect on job satisfaction. Individuals with high neuroticism demonstrate poorer emotional stability and are more prone to experiencing negative emotions such as anxiety, depression, and tension. Within the environment of designated infectious disease hospitals—where staff face prolonged exposure to infection risks, pressures of critical care, and potential social stigma—such physicians are more likely to internalize work stress as psychological burdens, thereby substantially diminishing their job satisfaction. Previous research has shown that neuroticism can diminish job satisfaction by increasing burnout within professional quality of life [28]. It is noteworthy that within the specific healthcare setting of this study, the enhancement of job satisfaction attributable to the social engagement fostered by extraversion may be less pronounced than that derived from traits more intrinsically linked to core work tasks, such as conscientiousness and agreeableness. Although extraversion exhibited a relatively modest direct effect on job satisfaction in this research, some literature continues to support its positive influence. Extraversion is typically associated with enthusiasm, self-confidence, sociability, and positive affect. A longitudinal study found that increases in extraversion from adolescence to early adulthood predicted higher career satisfaction and job satisfaction [29]. In Nigerian employee studies, extraversion also exhibited a positive correlation with job satisfaction [30]. However, this relationship may be moderated by task characteristics and environmental factors. In certain medical settings requiring high concentration and independent work, the advantages of extraversion may be less pronounced than in socially intensive occupations. Nevertheless, fostering a supportive work environment with robust social interaction may help leverage the strengths of extraversion, thereby exerting a positive influence on job satisfaction.

### Mediating mechanism of negative coping strategies

This study validated through structural equation modeling that negative coping strategies mediate the relationship between personality traits and job satisfaction. This indicates that personality traits not only directly shape physicians’ satisfaction but also exert an indirect influence by affecting the coping strategies they employ when facing stress.

Negative coping styles, such as avoidance, emotional venting, and self-blame, have been extensively demonstrated to serve as crucial mediating variables linking negative psychological experiences to adverse adaptive outcomes. For instance, among community correctional individuals, feelings of relative deprivation significantly predict future risky behaviours, with this relationship partially mediated by negative coping styles [31]. Similarly, among postgraduate students, childhood psychological maltreatment increases cyberbullying behaviour by amplifying negative coping styles and trait anxiety [32]. Collectively, these studies demonstrate that when individuals face stress or adverse circumstances, a tendency towards negative coping strategies exacerbates psychological distress and leads to more detrimental behavioural or emotional outcomes.

This study indicates that the indirect effect of neuroticism on job satisfaction via negative coping is -0.013. Neuroticism not only directly reduces satisfaction but also prompts individuals to adopt more negative coping strategies (such as avoiding problems, self-blame, and emotional venting). These ineffective coping mechanisms further exacerbate occupational burnout and psychological exhaustion, creating a vicious cycle of “stress → negative coping → decreased satisfaction.” Conversely, conscientiousness, agreeableness, openness, and extraversion are negatively correlated with negative coping, meaning physicians with these traits employ negative coping strategies less frequently. Highly conscientious physicians tend to adopt problem-focused coping, proactively addressing difficulties; those with high agreeableness excel at seeking social support; those with high openness demonstrate flexibility in adjusting cognition and strategies; and those with high extraversion typically possess stronger emotional expression and positive affective experiences. These positive tendencies reduce the use of negative coping, thereby exerting a positive indirect promotion effect on job satisfaction. Research also indicates that highly neurotic individuals are more inclined toward emotion-focused and avoidance coping, while high levels of extraversion, conscientiousness, agreeableness, and openness are significantly associated with more positive and constructive coping tendencies [33, 34]. Consequently, negative coping, as a maladaptive strategy, struggles to effectively resolve stress-related issues, ultimately leading to reduced job satisfaction.

Based on the findings of this study, management strategies to enhance job satisfaction among physicians at designated infectious disease treatment centres should be advanced through coordinated efforts at both the individual intervention and organisational support levels. At the individual level, assessments of personality traits and coping styles should be integrated into routine psychological support systems. For clinicians exhibiting high neuroticism, targeted interventions in stress management and emotional regulation should be implemented. Cognitive behavioural training can assist them in identifying and modifying automatic negative coping patterns, such as transforming problem avoidance into step-by-step problem-solving, and shifting excessive self-blame towards self-acceptance and emotional regulation [35]. Concurrently, physicians exhibiting high levels of conscientiousness, agreeableness, openness, and extraversion should be provided with role assignments and development opportunities that leverage their personality strengths, thereby enhancing their professional fulfilment and work engagement. At the organisational level, the critical imperative lies in systematically cultivating a work environment and organisational culture that supports positive coping. This involves refining remuneration incentives and promotion mechanisms, clarifying role boundaries, expanding clinical decision-making participation, strengthening team communication, and providing timely psychological support. Such measures enhance physicians’ sense of control, meaning, and belonging, thereby fundamentally promoting the adoption of positive coping strategies, reducing long-term stress burdens, and ultimately elevating job satisfaction and professional commitment [36].

This empirical study reveals the operational mechanisms linking personality traits, negative coping styles, and job satisfaction among physicians at designated infectious disease treatment centres. Findings indicate that personality characteristics directly influence job satisfaction while also exerting indirect effects through coping mechanisms. Neuroticism significantly diminishes job satisfaction by amplifying negative coping, whereas conscientiousness, agreeableness, openness, and extraversion exert stable positive effects on job satisfaction. The findings suggest that within the high-pressure, high-risk context of infectious disease control, clinicians’ occupational mental health constitutes not merely an individual concern but also a vital organisational resource influencing healthcare institutions’ resilience and emergency response capacity. Consequently, healthcare institutions and policymakers should adopt a dual-pronged approach combining individual psychological interventions with organisational support systems. By strengthening emotional regulation and coping skills training, optimising human resource allocation, and refining institutional safeguards, they can enhance physicians’ job satisfaction and occupational wellbeing. This will ultimately bolster the overall stability and public health response capacity of designated infectious disease treatment facilities.

### Limitations and future research directions

Firstly, the cross-sectional study design limits causal inference; future longitudinal or intervention studies are required to further validate causal pathways between variables. Secondly, as the sample originates from designated medical institutions in a single region, the generalisability of findings requires validation across multiple regions and diverse healthcare settings. This study focused on individual-level factors with insufficient attention to organisational environments and situational variables. Subsequent research should develop integrated models combining personality traits, coping strategies, and organisational support. More targeted mechanism and intervention studies should address the unique stressors faced by infectious disease-designated medical institutions, thereby providing stronger theoretical and empirical foundations for implementing precise psychological support and optimising human resource management.

## Conclusion

This study found that conscientiousness, agreeableness, openness, and extraversion exerted significant positive effects on job satisfaction, whereas neuroticism produced a significant negative impact. Negative coping styles mediated this relationship partially, particularly as neuroticism further diminished job satisfaction by increasing negative coping. The findings theoretically enrich the research framework linking personality traits to occupational psychological outcomes. Practically, they suggest healthcare institutions should enhance physicians’ job satisfaction and professional wellbeing by optimising coping strategies and organisational support systems.

## Data Availability

The data for this study were derived from an original questionnaire that collected 8,071 valid responses. Due to respondent privacy concerns and ethical agreements, the raw data are not publicly available. To support academic verification, we implement conditional data sharing: during the review process, anonymized data may be provided to the journal; after publication, researchers can request the data by sending an email to the author’s email address 1178708407@qq.com.Upon approval by the ethics committee and execution of a data use agreement, an ethically compliant data subset will be provided.

## Abbreviations

CBF–PI–B: Chinese Big Five Personality Inventory Brief Version
SCSQ: Simplified Coping Style Questionnaire
MSQ: Minnesota Satisfaction Questionnaire
CI: Confidence interval
P: Probability
SEM: Structural equation modeling
χ2/DF: Chi-square to degrees of freedom ratio
CFI: Comparative fit index
TLI: Tucker-Lewis index
IFI: Incremental fit index
GFI: Goodness-of-fit index
AGFI: Adjusted Goodness-of-Fit index
RMSEA: Root mean square error of approximation

## References

[1] Ji W, Liu Y, Sun Q, Wu D, Liu T, Sun P. Burnout and job stress in healthcare professionals: a single-centre cross-sectional study in an East China tertiary hospital after COVID-19 policy adjustment. BMJ Open. 2025;15(9):e099854. 41033772 10.1136/bmjopen-2025-099854 PMC12496090

[2] Shanafelt TD, West CP, Dyrbye LN, Trockel M, Tutty M, Wang H, Carlasare LE, Sinsky C. Changes in burnout and satisfaction with Work-Life integration in physicians during the first 2 years of the COVID-19 pandemic. Mayo Clin Proc. 2022;97(12):2248–58. 36229269 10.1016/j.mayocp.2022.09.002 PMC9472795

[3] Boone A, Lavreysen O, De Vries N, De Winter P, Mazzucco W, Matranga D, Maniscalco L, Miceli S, Savatteri A, Kowalska M, et al. Retaining healing hands: A transnational study on job retention interventions for the healthcare workforce. Qual Health Res. 2024;34(13):1351–66. 38857417 10.1177/10497323241254253

[4] Kang W, Malvaso A. Associations between personality traits and areas of job satisfaction: pay, work itself, security, and hours worked. Behav Sci (Basel). 2023;13(6). 10.3390/bs13060445 PMC10295380 37366697

[5] Opoku Mensah A, Koomson S. Openness to experience moderates psychological contract breach–job satisfaction tie-in. PSU Res Rev 2021. Ahead-of-print.

[6] Frisone F, Sicari F, Settineri S, Merlo EM. Clinical psychological assessment of stress: A narrative review of the last 5 years. Clin Neuropsychiatry. 2021;18(2):91–100. 34909024 10.36131/cnfioritieditore20210203 PMC8629067

[7] Zhou H, Peng J, Wang D, Kou L, Chen F, Ye M, Deng Y, Yan J, Liao S. Mediating effect of coping styles on the association between psychological capital and psychological distress among Chinese nurses: a cross-sectional study. J Psychiatr Ment Health Nurs. 2017;24(2–3):114–22. 28233374 10.1111/jpm.12350

[8] Wang Y, Wang P. Perceived stress and psychological distress among Chinese physicians: the mediating role of coping style. Med (Baltim). 2019;98(23):e15950. 10.1097/MD.0000000000015950 PMC6571215 31169719

[9] Ringwald WR, Nielsen SR, Mostajabi J, Vize CE, van den Berg T, Manuck SB, Marsland AL, Wright AGC. Characterizing stress processes by linking big five personality states, traits, and day-to-day stressors. J Res Pers. 2024;110. 10.1016/j.jrp.2024.104487 PMC11067701 38708104

[10] Shu H, Ren M, Wang H, Sun X, Feng D. The relationship between nurses’ occupational stress and quality of life: A moderated mediation model. J Shandong Univ (Medical Edition). 2024;62(01):89–94.

[11] Yao Y, Tang J, Li Z, Chen S, Du H, Lu L. Social support and psychological capital mediate the effect of personalities on the mental health of professional staff in China during COVID-19 pandemic. Psychol Res Behav Manag. 2024;17:3443–53. 39385810 10.2147/PRBM.S475165 PMC11463178

[12] Wang M, Dai X, Yao S. Preliminary development of the Chinese big five personality questionnaire I: theoretical framework and reliability analysis. Chin J Clin Psychol. 2010;18(05):545–8.

[13] Xie Y. Preliminary study on the reliability and validity of the simplified coping style scale. Chin J Clin Psychol. 1998(02):53–4.

[14] Weiss DJ, Dawis RV, England GW. Manual for the Minnesota satisfaction questionnaire. Minn Stud Vocat Rehabilitation. 1967;22:120–120.

[15] Wu J, Zhou J. How the configurations of job autonomy, work-family interference, and demographics boost job satisfaction: an empirical study using FsQCA. Asian Bus Manag. 2022;21(4):547–68. 40477184 10.1057/s41291-020-00138-8 PMC7565726

[16] Shiga T, Hifumi T, Hagiwara Y, Otani N, Tanaka H, Nakano M, Kuroda Y. Career satisfaction among acute care resident physicians in Japan. Acute Med Surg. 2022;9(1):e779. 36051448 10.1002/ams2.779 PMC9420170

[17] Saberi M, Davoudi-Monfared E, Naderi M. Satisfaction of physicians working in a referral hospital in Tehran, Iran in 2019. Hosp Practices Res. 2020;5:70–4.

[18] Lodin HM, Bersoux S, Pannala R, Mi L, Vegunta S. Primary care delivery perceptions and their associations with physician and patient gender. J Community Health. 2023;48(4):711–7. 36976390 10.1007/s10900-023-01211-x

[19] Zhang P, Liu L, Liao X, Wu J, Yang Z, Zhang Y. A study on the job satisfaction and influencing factors of general practitioners in primary healthcare institutions. Chin Gen Pract. 2025;28(07):869–74.

[20] Huang J, Miao Y, He X, Jiang D, Liang Y. A systematic review of work satisfaction and influencing factors among general practitioners in China. Chin Gen Pract. 2025;28(10):1220–7.

[21] Shfiee M, Ahmadi Y, Azizi M, Pishgooie A, Afaghi E. Investigating the relationship between personality traits and job satisfaction in Iranian nurses during the COVID-19. Health Emergencies Disasters Q. 2024;10:29–36.

[22] Ren C, Ma X, Xie H, Wang Y. A Study on the impact of physician-nurse collaboration on nurses’ work engagement. Chin J Nurs Manage. 2016;16(06):754–8.

[23] Redelmeier DA, Najeeb U, Etchells EE. Understanding patient personality in medical care: Five-Factor model. J Gen Intern Med. 2021;36(7):2111–4. 33506393 10.1007/s11606-021-06598-8 PMC7840072

[24] Ammi M, Fooken J, Klein J, Scott A. Does doctors’ personality differ from those of patients, the highly educated and other caring professions? An observational study using two nationally representative Australian surveys. BMJ Open. 2023;13(4):e069850. 37094898 10.1136/bmjopen-2022-069850 PMC10186421

[25] Liu M, Cai J, Chen H, Shi L. Association of personality traits with life and work of medical students: an integrative review. Int J Environ Res Public Health. 2022;19(19). 10.3390/ijerph191912376 PMC9566667 36231679

[26] Louwen C, Reidlinger D, Milne N. Profiling health professionals’ personality traits, behaviour styles and emotional intelligence: a systematic review. BMC Med Educ. 2023;23(1):120. 36803372 10.1186/s12909-023-04003-y PMC9938999

[27] Stienen MN, Scholtes F, Samuel R, Weil A, Weyerbrock A, Surbeck W. Different but similar: personality traits of​ surgeons and internists-results of a cross-sectional observational study. BMJ Open. 2018;8(7):e021310. 29982214 10.1136/bmjopen-2017-021310 PMC6045716

[28] Li WW, Xie G. Personality and job satisfaction among Chinese health practitioners: the mediating role of professional quality of life. Health Psychol Open. 2020;7(2):2055102920965053. 33178439 10.1177/2055102920965053 PMC7592332

[29] Hoff KA, Einarsdóttir S, Chu C, Briley DA, Rounds J. Personality changes predict early career outcomes: discovery and replication in 12-Year longitudinal studies. Psychol Sci. 2021;32(1):64–79. 33226888 10.1177/0956797620957998

[30] Jarrar Y, Ammar L, Nweke G, Horoub I, Aderibigbe A. Organizational communication satisfaction as a moderator between the big five personality traits and job satisfaction among employees in Nigeria. Front Psychol. 2024;15:1339305. 40692825 10.3389/fpsyg.2024.1339305 PMC12278842

[31] Tang L, Xu T, Ma J. Psychological predictors of future dangerous behavior among probationers: evidence from judicial bureaus in four provinces in China. Front Psychol. 2025;16:1593698. 41235263 10.3389/fpsyg.2025.1593698 PMC12604979

[32] Shen Y. The relationship between childhood psychological abuse and cyberbullying behavior among graduate students: the mediating role of negative coping style and trait anxiety. Front Psychiatry. 2024;15:1497407. 39676913 10.3389/fpsyt.2024.1497407 PMC11638203

[33] Carver CS, Connor-Smith J. Personality and coping. Annu Rev Psychol. 2010;61:679–704. 19572784 10.1146/annurev.psych.093008.100352

[34] Gashi D, Gallopeni F, Imeri G, Shahini M, Bahtiri S. The relationship between big five personality traits, coping strategies, and emotional problems through the COVID-19 pandemic. Curr Psychol. 2022:1–10. 10.1007/s12144-022-03944-9 PMC9660183 36406846

[35] Luo A, McAloon J. Potential mechanisms of change in cognitive behavioral therapy for childhood anxiety: A meta-analysis. Depress Anxiety. 2021;38(2):220–32. 33225527 10.1002/da.23116

[36] Zhang S, Shi Y, Liu B, Wang H, Zhao X, Wang X, Sun T. Job demands and resources and their relationship with satisfaction and thriving at work in a sample of Chinese doctors: a cross-sectional study. BMJ Open. 2021;11(11):e045745. 34845064 10.1136/bmjopen-2020-045745 PMC8633991

